# Consanguineous Marriages and Dental Anomalies: A Cross-Sectional Analytical Study

**DOI:** 10.1155/2022/9750460

**Published:** 2022-04-06

**Authors:** Beenish Abbas, Sana Abbas, Saadia Muneer Malik, Majida Rahim, Muhammad Umair, Zohaib Khurshid

**Affiliations:** ^1^Department of Pedodontics, Foundation University College of Dentistry, Islamabad, Pakistan; ^2^Department of Anesthesiology, National University of Medical Science, Islamabad, Pakistan; ^3^Department of Oral Pathology, National University of Medical Science, Islamabad, Pakistan; ^4^Department of Oral Medicine, Foundation University College of Dentistry, Islamabad, Pakistan; ^5^Department of Prosthodontics and Implantology, College of Dentistry, King Faisal University, Hofuf, Saudi Arabia

## Abstract

**Objective:**

To determine the correlation between consanguineous marriages and dental anomalies. *Study Design*. A cross-sectional analytical study.

**Materials and Methods:**

This cross-sectional analytical multicentered study was carried out at Foundation University College of Dentistry after approval of the Ethical Research Committee (ERC) from September 2021 to November 2021 in Pakistan. All pediatric patients (4–10 years old) with dental malformations undergoing dental procedures or examinations and born with spontaneous vaginal delivery and uncomplicated cesarean section participated in the study. First, second, and third-degree relatives' marriages of parents were used to define consanguinity. The Basic Pay Scale was the reference of estimation of socioeconomic status. Participants with a history of orofacial trauma, pertinent parental history (infertility, hormonal treatment, or infectious diseases during pregnancy, conception with assisted reproductive techniques, prolonged complicated labor, premature deliveries, and twin births), and prolonged hospital admission immediately after birth were excluded from the study.

**Results:**

The mean maternal and paternal age was recorded to be 23.86 ± 5.4 and 27.07 ± 9.6, respectively, whereas the mean age of participants was 6.60 ± 1.67. There were 297 children with congenital abnormalities, with 203 (68.4%) males and 94 (31.6%) females. The prevalence of consanguineous marriage was found to be in 210 (70.7%) participants. Congenital dental anomalies correlation was found to be significant with consanguineous marriages (*p* value <0.001). Consanguineous marriages were more frequent in the lower socioeconomic group when compared with the middle and upper socioeconomic groups, respectively (74.7% vs. 8.1% vs. 17.2%, *p* value 0.007).

**Conclusion:**

Congenital dental anomalies were significantly prevalent in consanguineous marriages with greater incidence in lower socioeconomic groups. Consanguineous marriages have the propensity to transmute population conformation, but due to religious and social beliefs, literature is hesitant to ascribe congenital dental anomalies persuasive relevance with consanguinity.

## 1. Introduction

Consanguinity is a derivative of a Latin word that refers to “Con” meaning “Common” and “Sanguineous” meaning “Blood” [1]. There is an exaggerated risk of autosomal recessive disorders transmission generation after generation due to increased likelihood of carrier finding a similar disorder unless related. Owing to 12.5% genes similarities between first-degree cousins, the prevalence of homozygous gene loci is estimated to be 6.25%, hence rendering a risk of 1 in 20 when compared to 1 in 40 in the general population [2, 3].

Pakistan and India are among the countries reporting the highest rate of consanguinity with the incidence of 73% and 5–60%, respectively. It has been estimated recently that 2.34% of total deaths in Pakistan are attributable to congenital anomalies. Here, the high incidence of CA has been attributed to various factors, such as maternal malnutrition, inadequate prenatal care, poor socioeconomic setup, rural origin, and a high rate of consanguinity [4].

Syria and Lebanon warranted an obligation of premarital screening to acquire marriage certificates, whereas South Asian communities lag such level of awareness and counseling protocols [5, 6]. Cosmic disparity of preferential consanguineous marriages recorded in Europe versus West Asia, south India, and North Africa represents 1% and 20–50%, respectively [7, 8].

Tooth eruption and development involves an eccentric and distinctive pattern under influence of genetic and environmental factors; hence, any interruption in eruptive development of tooth germ from the alveolar crypt of the jaw towards functional disposition in oral cavity results in dental anomalies [9, 10].

Various dental anomalies such as amelogenesis imperfecta, dentinogenesis imperfecta, molar incisor hypomineralization, dens invaginatus, and double teeth can be diagnosed by detailed intraoral and extraoral examination and investigations like orthopantomography, periapical radiographs, and cone beam compute tomography. However, during recent year, techniques using less ionizing radiations have been suggested to limit biological damage specially in young children [11].

The rationale of this study was to track down the ubiquity of consanguineous marriages and dental anomalies owing to the scarcity of literature on the subject, although blood dyscrasias and mental conditions have established definitive association with consanguineous marriages.

## 2. Materials and Methods

This cross-sectional analytical multicentered study was carried out at Foundation University College of Dentistry after approval of the Ethical Research Committee from September 2021 to November 2021 in Pakistan.

The minimum sample size calculated for the study was (*n* = 254), where the prevalence of dental anomalies was considered to be 20.9% with a 95% confidence level and 5% margin of error as reported by Lagana et al. [12].

Informed written consent was obtained by the parents of the participants. All pediatric patients (4–10 years old) with dental malformations undergoing dental procedures or examinations and born with spontaneous vaginal delivery and uncomplicated cesarean section participated in the study. First, second, and third-degree relatives' marriages of parents were used to define consanguinity. The Basic Pay Scale was the reference of estimation of socioeconomic status. Participants with a history of orofacial trauma, pertinent parental history (infertility, hormonal treatment, radiation exposure, or infectious diseases during pregnancy, conception with assisted reproductive techniques, prolonged complicated labor, premature deliveries, and twin births), and prolonged hospital admission immediately after birth were excluded from the study. The history of congenital malformations in other offsprings and members of their family and parental consanguinity was obtained by interviewing the child's mother or father whoever accompanied and details endorsed accordingly. Pediatrician records were consulted to define syndromes if any.

Data were entered and analyzed by the data management software IBM SPSS (version 23.0). The descriptive statistics for the categorical variable were presented as frequency and percentage, while the mean and standard deviation were reported for continuous variables. The categorical groups were compared by using the chi-square test. A *p* value of ≤0.05 endorsed to be statistically significant.

## 3. Results

The mean maternal and paternal age was recorded to be 23.86 ± 5.4 and 27.07 ± 9.6, respectively, whereas the mean age of participants was 6.60 ± 1.67. There were 297 children with congenital abnormalities, with 203 (68.4%) males and 94 (31.6%) females. The prevalence of consanguineous marriage was found to be in 210 (70.7%) participants. Congenital dental anomalies correlation was found to be significant with consanguineous marriages (*p* value <0.001). Frequency of recorded anomalies was Goldenhar syndrome (dental malocclusion) 1 (0.3%), Bardet–Biedl syndrome (hypodontia, high-arched palate, crowding, microdontia, and shortened roots) 2 (0.7%), Steven–Johnson syndrome 2 (0.7%), ligneous periodontitis 2 (0.7%), Marfan syndrome (periodontitis) 2 (0.7%), macrodontia 3 (1.0%), concrescence 3 (1.0%), arrested root development 3 (1.0%), dens in dente 3 (1.0%), supernumerary teeth 3 (1.0%), supplemental teeth 3 (1.0%), mitochondrial leukodystrophy 3 (1.0%), oligodontia 3 (1.0%), accessory roots 6 (2.0%), germination 6 (2.0%), xeroderma pigmentosa (periodontitis and maxillary enamel hypoplasia) 6 (2.0%), Down syndrome (periodontitis) 7 (2.4%), ectopic eruption 8 (2.7%), genetic hypoplasia 8 (2.7%), congenital rubella syndrome (narrow maxillary arch and missing teeth) 9 (3.0%), talon's cusp 9 (3.0%), dens evaginatus 9 (3.0%), fusion 9 (3.0%), microcephaly (enamel defects and microdents) 12 (4.0%), microdontia 12 (4.0%), cerebral palsy 13 (4.4%), delayed eruption 15 (5.1%), hypodontia 21 (7.1%), root dilaceration 30 (10.1%), taurodontism 30 (10.1%), and hyperdontia 54 (18.2%) and is given in Table 1.

**Figure 1:**
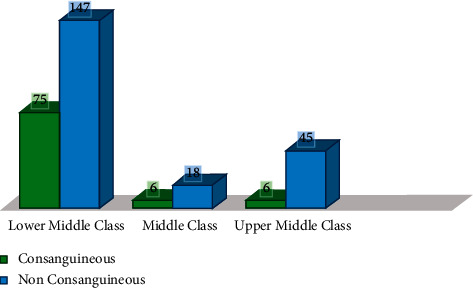
Association of consanguineous marriages and socioeconomic status.

Consanguineous marriages were more frequent in the lower socioeconomic group when compared with middle and upper socioeconomic group, respectively (74.7% vs. 8.1% vs. 17.2%, *p* value 0.007), as given in Table 2 and Figure 1, first cousin 252 (85%), second cousin 19 (6.5%), and third cousin 26 (8.5%).

## 4. Discussion

This cross-sectional analytical study was formulated to emphasize the disease burden caused by conformist beliefs in a third-world country with poorly resourced medical and healthcare facilities. Unfortunately, correlation with congenital syndromes and consanguineous marriages had been studied and pondered to some extent, but curiosity in the subject is still lacking in the field of dentistry. The thorough analysis in this study elaborated that the prevalence of consanguineous marriage was found to be in 210 (70.7%) participants with significant relevance with congenital dental anomalies (*p* value <0.001).

Consanguineous marriages were more frequent in the lower socioeconomic group when compared with the middle and upper socioeconomic group, respectively (74.7% vs. 8.1% vs. 17.2%, *p* value 0.007). This statistical fact corresponds with a lower literacy level of 58% in Pakistan, in addition to conservative approaches and lack of fair and square discussion before the institution of marital relationship [13].

Bagci N et al. evaluated nonsyndromic developmental dental anomalies based on clinical and radiological analyses and, additionally, association with self-narrated systemic ailments in 880 offspring of consanguineous and nonconsanguineous couples with an age limit of 16 years or greater with further subdivision into two batches of study (consanguineous marriages) and control (nonconsanguineous marriages) groups.

There was a statistically significant relationship between the consanguineous marriage and DDA types. The study proposed a significant correlation between consanguineous marriage and developmental dental anomalies in a particular size (microdontia) and morphological (dilaceration and taurodontism). Hence, results were coherent with our research analysis where taurodontism 30 (10.1%) and microdontia were seen in 12 (4%) participants [14].

Khan et al. carried out a cross-sectional analysis on a sample size of 2000 participants via a validated questionnaire of the association of consanguinity and dental developmental anomalies in 6–9-year-old children born out of consanguineous and nonconsanguineous marriages. Fusion and nonsyndromic supernumerary teeth (*p* value < 0.001) and microdontia (*p* value 0.002) represented a significant association with consanguinity [15].

Bardet–Biedl autosomal recessive syndrome involving cilia function and nineteen genes' mutations defined by G. Bardet and A. Biedl in the 1920s, although thin on ground, represents the ubiquitous relationship with consanguinity. This disease involves a multitude of clinical manifestations such as renal dysfunction, obesity, retinal dystrophy, polydactyly, learning disability, cognitive defects, genital, cardiac, and dental anomalies.

Oral and dental aberrations include hypodontia, high-arched palate, crowding, microdontia, and shortened roots; however, they are significant to differentiate from Alstrom and McKusick–Kaufman syndromes. Our cohort exhibited 2 (0.7%) patients with this rarity with a significant association with consanguineous marriages (*p* value < 0.001) [16].

Children born from consanguineous and nonconsanguineous marriage represent 1/20 and 1/40 prevalence of anomalies, respectively. Hence, scholarly notables should play role in education and awareness of the masses. The specific pattern of the consanguineous marriages depends upon the traditional rituals and ethnic faith, most prominent being between first cousins 85%, as elucidated by this study results as well [17, 18].

The sky-scraping practices of consanguineous marriages in our community are attributable to social practices, ignorance of the general population about associated adversaries, and reluctance to discuss such momentous issues. A matter of concern is that Pakistan ranked 149^th^ among 179 countries in 2015 on the Maternal Mortality Ratio Index, which is somehow pertinent to consanguineous marriages and congenital malformations as well [19, 20]. Perveen et al. conducted a similar study in tertiary care institute at Karachi and the lion's share turned out to be neural tube defects widely attributable to consanguinity [21].

Apotheosis of this study states consanguinity to be the highest attribute for genetic dental malformations. Since we have nearly the highest inbreeding coefficient and scarcity of epidemiological research work, therefore, it is imperative to incorporate genetic and marital counseling in addition to vigilant diagnosis and screening maneuvers. Pakistan has the lofty occurrence of congenital and hereditary anomalies predominantly attributable to consanguinity, early marriages, and extended families with weak healthcare infrastructure to encounter such divergent and challenging health conditions. Moreover, the majority of the population has financial resources to barely meet necessities of life, living in rural areas with limited access to modern healthcare assets, and hence cannot meet the financial burden of genetically and chronically ill children. Therefore, healthcare and social agencies should come into play for the education and awareness of the general public with the incorporation of religious experts' guidance.

## 5. Conclusion

Congenital dental anomalies were significantly prevalent in consanguineous marriages with greater incidence in the lower socioeconomic groups. Consanguineous marriages have the propensity to transmute population conformation, but due to religious and social beliefs, literature is hesitant to ascribe congenital dental anomalies persuasive relevance with consanguinity.

## Figures and Tables

**Table 1:** Association of consanguineous marriages and dental anomalies.

Dental anomaly	*N* (%)
Goldenhar syndrome (dental malocclusion)	1 (0.3)
Ligneous periodontitis	2 (0.7)
Bardet–Biedl syndrome	2 (0.7)
SJ syndrome	2 (0.7)
Marfan syndrome	2 (0.7)
Arrested root development	3 (1.0)
Concrescence	3 (1.0)
Macrodontia	3 (1.0)
Mitochondrial leukodystrophy	3 (1.0)
Oligodontia	3 (1.0)
Supernumerary teeth	3 (1.0)
Supplemental teeth	3 (1.0)
Dens in dente	3 (1.0)
Accessory roots	6 (2.0)
Xeroderma pigmentosa (periodontitis and maxillary enamel hypoplasia)	6 (2.0)
Germination	6 (2.0)
Down syndrome	7 (2.4)
Ectopic eruption	8 (2.7)
Genetic hypoplasia	8 (2.7)
Congenital rubella syndrome	9 (3.0)
Dens evaginatus	9 (3.0)
Fusion	9 (3.0)
Talon's cusp	9 (3.0)
Microcephaly (enamel defects and microdents)	12 (4.0)
Microdontia	12 (4.0)
Cerebral palsy	13 (4.4)
Delayed eruption	15 (5.1)
Hypodontia	21 (7.1)
Taurodontism	30 (10.1)
Root dilaceration	30 (10.1)
Hyperdontia	54 (18.2)

^*∗*^Significant *p* value <0.001 (dental anomaly vs. consanguineous marriage).

**Table 2:** Association of consanguineous marriages and socioeconomic status.

		Consanguineous marriages	
		No	Yes
Socioeconomic profile	Lower middle class	75 (33.8%)	147 (66.2%)
	Middle class	6 (25.0%)	18 (75.0%)
	Upper middle class	6 (11.8%)	45 (88.2%)

^*∗*^Significant *p* value 0.007.

## Data Availability

The dataset used to support the findings of this study is available from the corresponding author upon request.
